# Mindfulness and Relaxation-Based Interventions to Reduce Parental Stress, Anxiety and/or Depressive Symptoms in the Neonatal Intensive Care Unit: A Systematic Review

**DOI:** 10.1007/s10880-022-09902-8

**Published:** 2022-08-19

**Authors:** Kristin Harrison Ginsberg, Jane Alsweiler, Mohsen Alyami, Anna Serlachius

**Affiliations:** 1 Department of Psychological Medicine, School of Medicine, University of Auckland, Building 507, Level 3, 22-30 Park Avenue, Grafton, Auckland, 1023 New Zealand; 2 Department of Paediatrics: Child and Youth Health, School of Medicine, University of Auckland, Auckland, New Zealand; 3 Department of Psychological Medicine, School of Medicine, University of Auckland, Auckland, New Zealand

**Keywords:** Parents, NICU, Stress, Mindfulness, Relaxation

## Abstract

Parents with infants in the neonatal intensive care unit (NICU) experience high levels of stress, anxiety, and depression. Mindfulness and relaxation-based interventions are effective in reducing distress in the general postpartum population. The aim of this systematic review was to evaluate whether mindfulness and/or relaxation-based interventions reduce stress, anxiety, and depression in NICU parents. A total of five studies met the inclusion criteria and were assessed for quality using the Downs & Black Checklist. The most consistent results in this review suggest that mindfulness and/or relaxation-based interventions may be effective at reducing anxiety symptoms in NICU parents, with moderate to large effect sizes, and show promise in reducing depressive symptoms. The findings show limited potential benefits on parental stress. Methodological weaknesses, heterogeneous intervention factors (including format and length), and varying participant adherence hinder the ability to make strong conclusions. Directions for future research are discussed.

## Introduction

Worldwide, approximately 15 million infants are born preterm each year (Liu et al., 2016), and an estimated 50% or more may require care in a neonatal intensive care unit (NICU), a specialist service that is designed and equipped to care for very small or very unwell infants (Phaloprakarn et al., 2015). Parents of infants in the NICU experience increased stress due to a range of factors, including the NICU environment (Turner et al., 2015), disruptions in the parental role, feelings of powerlessness (Lean et al., 2018), witnessing distressing medical procedures performed on their infant (Obeidat et al., 2009), and logistical challenges around finances, childcare for older siblings, and time management (Turner et al., 2015).

Subsequently, parents of infants in the NICU experience higher rates of anxiety, depression, acute stress disorder, and post-traumatic stress disorder (PTSD) than the general postpartum population (Lean et al., 2018; Shaw et al., 2014; Turner et al., 2015). Prevalence studies in the NICU have found striking differences in these rates: 24.7% of NICU mothers experienced anxiety compared to 7.4 to 8.7% in the general maternal population (Lotterman et al., 2019); 39% of NICU parents had postpartum depression compared to 10 to 15% of non-NICU parents (Lefkowitz et al., 2010); and 18.5% of NICU parents met criteria for PTSD compared with 4% in the broader adult population (Yildiz et al., 2017). There are also longer-term adverse effects of the NICU experience after discharge, including impaired parenting and parent–child dyad disruptions in bonding and attachment (Lean et al., 2018).

### Interventions for NICU Parents

A diverse range of psychological interventions have been developed to improve mental health outcomes for NICU parents. A 2019 systematic review and meta-analysis sought to assess the effectiveness of a wide range of interventions, from psychotherapy to acupuncture, among parents with infants in NICU (Sabnis et al., 2019). This review found that family-centered care was associated with the most significant reduction in parental distress in the NICU, and it also noted that alternative/complementary interventions, including relaxation and meditation, showed early promise (Sabnis et al., 2019). This conclusion was similar to a previous review that focused on maternal mental health in the NICU, which noted that mindfulness-based strategies may be useful for reducing depression and anxiety symptoms (Mendelson et al., 2017).

### The Mind–Body Medicine Framework

Mindfulness-based interventions (MBIs) and relaxation-based interventions are considered similar but different components of mind–body medicine (Kabat-Zinn, 2005), an evidence-based framework focused on the interactions between the mind, body, and behavior (Luberto et al., 2020). However, the terms mindfulness and relaxation have been used interchangeably in clinical intervention research in the last four decades in part because the two approaches use a combination of overlapping skills, such as deep breathing and visualization, in their often multi-modal interventions (Luberto et al., 2020). Both approaches have been found to be effective at reducing symptoms of anxiety and depression, among other mental health conditions (Jain et al., 2007; Luberto et al., 2020).

Despite the similarities, MBIs and relaxation-based interventions differ in notable theoretical ways. MBIs seek to increase distress tolerance and teach acceptance (not change) of emotions and physical sensations (Kabat-Zinn, 2005). Relaxation techniques, however, actively attempt to reduce distress (Rausch et al., 2006). Achieving both of these goals can be useful in highly stressed populations, and that may be why mindfulness and relaxation-based skills are often combined into multi-modality intervention programs in medical settings (Luberto et al., 2020). Given the changing definition of these terms over time, both MBIs and relaxation-based interventions were included in this systematic review.

### Mindfulness-Based Interventions (MBIs)

MBIs use the core tenets of mindfulness, which seek to intentionally increase awareness of the present moment, non-judgmentally, and sustain this attention over time (Kabat-Zinn, 2005). Stemming from Buddhist meditative practices, MBIs have been implemented in many clinical settings due in significant part to the success of the multi-week Mindfulness-Based Stress Reduction (MBSR) program, which utilizes a combination of meditation, breathing, and gentle movement components. Developed by Jon Kabat-Zinn and colleagues at the University of Massachusetts Medical School, MBSR is now delivered at more than 200 medical centers around the world (Niazi & Niazi, 2011) and has been shown to be effective in reducing symptoms of stress, PTSD, anxiety, depression, and chronic pain in adolescent and adult populations (Grossman et al., 2004).

### Relaxation-Based Interventions

Relaxation-based interventions are used to reduce the activation of the sympathetic nervous system (e.g., the “fight or flight” response) and increase the response of the parasympathetic nervous system (e.g., the “rest and digest” response) (Ma et al., 2017). Practices focus on decreasing arousal and increasing a sense of calm, often through slowing the breath, tensing and relaxing the muscles, or focusing the mind on positive mental experiences (Luberto et al., 2020).

Research into MBIs and relaxation-based interventions has grown exponentially in the last two decades, due in part to their relatively low-cost and ease of delivery (Perrier et al., 2020). Some of the most common techniques, which are often paired together in multi-modal intervention programmes, are detailed in brief below.

#### Deep Breathing

Diaphragmatic breathing, also referred to as deep breathing, focuses on contracting the diaphragm, deepening and slowing the rate of inhalation and exhalation, and expanding the abdomen (Ma et al., 2017). This type of breathing has been found to lead to reductions in anxiety, stress, and symptoms of PTSD (Colgan et al., 2016). Deep breathing can also be effective at improving cognitive functioning during stressful situations, such as university exams (Paul et al., 2007).

#### Progressive Muscle Relaxation (PMR)

PMR involves taking deep breaths while tightening, holding, and relaxing different muscle groups throughout the body in a progressive order, often beginning at the head and working down to the feet (Liu et al., 2020). Originally developed by Edmund Jacobson in 1938 to stimulate a relaxation response in the body (Hughes et al., 2013), PMR has been found to be effective at reducing anxiety symptoms and improving sleep in a wide range of studies and populations, including pregnant women (Rajeswari & Sanjeeva, 2019), cancer patients (Gok et al., 2019), and hospitalized COVID-19 patients (Liu et al., 2020).

#### Guided Imagery

Guided imagery uses external guidance to prompt internal visualization of images intended to improve positive affect (Hart, 2008; Rossman, 2000). Research has tested guided imagery with a broad range of populations, including adults diagnosed with a generalized anxiety disorder (Nguyen & Brymer, 2018), women with breast cancer (Kolcaba & Fox, 1999), and hospitalized psychiatric patients (Apostolo & Kolcaba, 2009) and reported reductions in symptoms of anxiety, depression, and stress, among other conditions.

### Rationale and Aim of Current Review

The effects of MBIs and/or relaxation-based interventions on NICU parents have not been broadly studied (Mendelson et al., 2017), and no systematic review has been published to date that focuses exclusively on this topic. However, there is a large body of evidence that supports these types of interventions to reduce symptoms of anxiety, depression, and stress in adult populations (Niazi & Niazi, 2011), and a small but growing group of literature showing support for NICU parents (Sabnis et al., 2019). Additionally, many existing intervention programs for NICU parents can be complex to deliver and costly to acquire (Sabnis et al., 2019). Mindfulness and relaxation-based interventions, however, may offer cost-effective alternatives (Saha et al., 2020), making them potentially more viable options in NICU settings with limited funding and staff resources.

Therefore, this systematic review assesses MBIs and relaxation-based interventions used in the NICU to reduce parental stress, anxiety, and depression. This review was undertaken to achieve the following aims: (1) to characterize the populations, interventions, delivery methods, and outcomes reported in peer-reviewed studies; (2) to estimate the overall effectiveness of interventions on parental stress, anxiety, and depression during NICU hospitalization and secondary outcomes such as breastfeeding measures; and (3) explore any differences in outcomes by delivery method (e.g., pre-recorded vs in-person).

## Methods

This review was performed per the Preferred Reporting Items for Systematic Reviews and Meta-Analyses (PRISMA) guidelines (Page et al., 2021) and registered with the International Prospective Register of Systematic Reviews (PROSPERO registration number 2021 CRD42021247715).

### Study Design

MedLine(OVID), PsycINFO, Embase, SCOPUS, Cochrane Library, and CINAHL Plus were searched using search terms (and synonyms) related to the intervention and setting including: mindfulness, relaxation therapy, guided imagery, meditation, Acceptance and Commitment Therapy (ACT), MBSR, NICU, intensive care, preterm, neonate. (See Appendix for the full search strategy). The search was restricted to studies published in English and in peer-reviewed journals, with no limits on the date of publication. No terms for the specified population or outcomes were included to ensure all possible studies were identified. Bibliographies of included studies, review papers, and conference abstracts were hand-searched to identify potential additional items. The database search was conducted between March 22 and March 31, 2021.

### Study Selection and Eligibility Criteria

All published studies (randomized trials and observational cohort studies including pre-post study designs) were included that were conducted in a NICU in any country and (1) reported one or more of this review’s primary and/or secondary outcomes using standardized measurement tools and (2) compared parents who received mindfulness or relaxation-based intervention with parents who received either another intervention or standard care or (3) studies that delivered the experimental intervention and conducted pre- and post-intervention measurements on the same group of parents. Case series, conference abstracts, and qualitative studies were excluded.

Mindfulness or relaxation-based interventions were defined as those using a psychological approach in order to reduce psychological stress, increase awareness of the present moment, or as defined by the study authors. Examples included deep breathing/diaphragmatic breathing, guided imagery, and progressive muscle relaxation. Multi-modal intervention programs that included mindfulness or relaxation skills and other components such as parenting education or psychological therapy were excluded. Interventions that involved non-psychological specialized treatments such as music therapy or art therapy were also excluded.

### Risk of Study Bias

Risk of bias and overall quality for each included study was assessed using the Downs and Black Checklist (D&B) (Downs & Black, 1998), designed for use with randomized and non-randomized intervention research. This checklist assesses the following domains for bias: reporting results, external validity, confounding factors, internal bias, and power analysis. Two researchers (KHG and MA) independently performed the risk of bias assessments. Conflicts were discussed and resolved by consensus. Scores were tallied to create a quality score with the ranges of excellent (26–28); good (20–25); fair (15–19); and poor (≤ 14) (Downs & Black, 1998).

### Outcomes

The primary outcomes were parental stress, anxiety, and/or depression. These outcomes, as defined by the study authors, were assessed through the use of a wide range of standardized screening tools. The secondary outcomes were breastfeeding measures defined by the quantity of expressed breast milk, exclusive breastfeeding on discharge from the NICU, or as defined by authors.

### Data Extraction

Search results were exported into Endnote V9 and duplicate articles were removed. The primary researcher (KHG) conducted the database search, and then records were screened by title, abstract, and full-text by two researchers (KHG and MA) for inclusion or exclusion using the software program Rayyan, a free, commonly used web-based tool recommended for the screening stage of systematic reviews (Kellermeyer et al., 2018). Disagreements about inclusion were resolved through a discussion between two researchers.

### Data Synthesis

Owing to study heterogeneity and the small sample of included studies, formal meta-analyses were not attempted. A narrative synthesis was conducted with the information presented in text and tables to describe the characteristics and reported the effectiveness of included studies. The narrative synthesis explored findings within and among included studies.

## Results

**Fig. 1:**
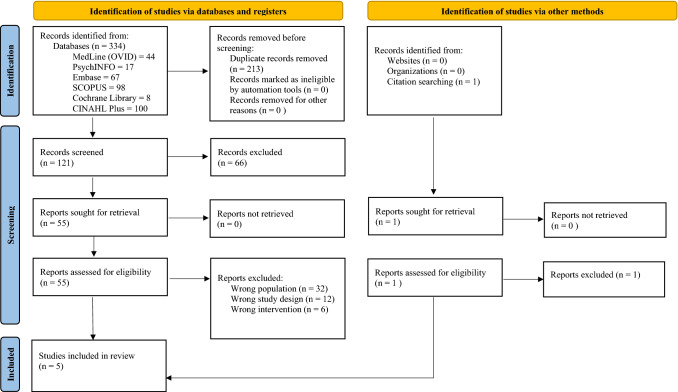
Study selection flow chart

Study selection is illustrated in Fig. 1. Through database searches and hand searches, 334 records were identified. Of those, 213 duplicates were removed. Of the remaining 121 records, title and abstract reviews excluded 66 articles, and an additional 52 were excluded after full-text review. Thus, five studies were included in this review.

### Characteristics of Included Studies

The five studies included in this review included 226 parents (Table 1), with individual study sample sizes that ranged from 20 to 71 parents. Overall, mothers made up the majority of the reported sample (more than 90%), with just one study including fathers (Marshall et al., 2019). Only three studies reported the race or ethnicity of participants (Howland et al., 2017; Marshall et al., 2019; Mendelson et al., 2018), and of those, the majority (61%) were Caucasian. The mean age of participants across all studies was 28.65 years old (SD = 1.66). The majority of studies were published in the last decade, from 2013 to 2019 (Dabas et al., 2019; Howland et al., 2017; Marshall et al., 2019; Mendelson et al., 2018), while one study was published in the 1980s (Feher et al., 1989). Four were conducted in the U.S. (Feher et al., 1989; Howland et al., 2017; Marshall et al., 2019; Mendelson et al., 2018) and one in India (Dabas et al., 2019).

**Table 1:** Characteristics of included studies

Author (Year)	Title	Study location	Type of intervention program	Participant characteristics	Study design	Comparison Type	Outcomes measured	Measurement time points	Measurement tools
Dabas et al. (2019)	Impact of audio assisted relaxation technique on stress, anxiety, and milk output among postpartum mothers on hospitalized neonates: A randomized controlled trial	India	Relaxation only (deep breathing, PMR, & gentle stretches)	57 mothers Age: CG: *M* = 29; IG: *M* = 30 No race or ethnicity reported	RCT	CG with standard care (28) or IG (29)	• Stress • Anxiety • Breast milk output	4 ± 2 days post-birth (baseline); 10 days post- enrolment	• PASS • PSS:NICU • Expressed breast milk volume
Feher et al. (1989)	Increasing breast milk production for premature infants with a relaxation/ imagery audiotape	USA	Relaxation only (guided imagery & PMR)	71 mothers Age: CG: *M* = 26.8; IG: *M* = 24.5 No race or ethnicity reported	RCT	CG with standard care (33) or IG (38)	• Breast milk volume • Breast milk cream %	3–5 days post-birth (baseline); 1 week after enrollment	• Expressed breast milk volume • Breast milk cream %
Howland et al. (2017)	Feasibility of a relaxation-guided imagery intervention to reduce maternal stress in the NICU	USA	Relaxation only (guided imagery)	20 mothers Age: *M* = 27.3 Race: White 60%; Other 20%; Black 5%; Asian 5%; Pacific Islander 5%; Native American 5% Ethnicity: Non-Hispanic 50%, Hispanic 50%	Pre-post feasibility study	Same group of mothers	• Stress • Depression • State anxiety • Maternal-infant responsiveness	"Shortly after birth" (baseline); 8-weeks after study enrollment	• PSS • CES-D • STAI • Maternal–Infant Response Instrument • Salivary cortisol levels
Marshall et al. (2019)	Mindfulness training among parents with preterm neonates in the neonatal intensive care unit: A pilot study	USA	Mindfulness only (including "relaxing sighs,” “calming phrases,"& meditation)	51 parents (26 mothers and 10 fathers from data available for 36 parents) Age: *M* = 29.2 Race: White: 69%, Black/African American: 22%, Unreported: 9%	Pre-post pilot study	Same group of parents	• Parental stress • Mindfulness	Within 2 weeks post-birth (baseline); 4 days prior to discharge	• PSS:NICU • CAMS-R • Self-report coping survey (created by study team)
Mendelson et al. (2018)	A mindfulness intervention to reduce maternal distress in neonatal intensive care: A mixed methods pilot study	USA	Mindfulness only (meditation)	27 mothers Age: *M* = 30.96 Race: White: 54.2%, African-American/Black: 41.7%, Asian or Pacific Islander: 4.2% Ethnicity: Not Hispanic/Latino: 87.5%, Hispanic/Latino: 12.5%	Pre-post pilot study	Same group of mothers	• Depression • Anxiety • Trauma symptoms • Sleep quality • NICU-related stress • Coping skills • Mindfulness skills • Maternal attachment to the infant • Self-compassion	Undefined baseline; 2 weeks post-enrollment	• PHQ-8 • GAD-7 • SASRQ • PSS-NICU • Brief COPE • PSQI • FFMQ • MIBS • Self-Compassion Scale

*RCT* randomized controlled trial, *PMR* progressive muscle relaxation, *CG* control group, *IG* intervention group, *PASS* Perinatal Anxiety Screening Scale, *PSS:NICU* Parental Stress Scale: Neonatal Intensive Care Unit, *CAMS-R* Cognitive and Affective Mindfulness Scale-Revised, *PHQ-8* Patient Health Questionnaire-8, *GAD-7* General Anxiety Disorder-7, *SASRQ* Standard Acute Stress Reaction Questionnaire, *COPE* Coping Orientation to Problems Experienced, *PSQI* Pittsburgh Sleep Quality Index, *FFMQ* Five Facets of Mindfulness Questionnaire, *MBIS* Mother–Infant Bonding Screening, *LOS* length of stay, *PMA* Postmenstrual age at discharge, *CES-D* Center for Epidemiologic Studies Depression Scale, *STAI* State-Trait Anxiety Inventory, *PSS* Perceived Stress Scale, *BDI-II*, Beck Depression Inventory, Second Edition, *BAI* Beck Anxiety Inventory

Half of these studies were labeled “pilot study” (Marshall et al., 2019; Mendelson et al., 2018) or “feasibility study” (Howland et al., 2017). Two of the studies were RCTs (Dabas et al., 2019; Feher et al., 1989).

### Risk of Bias Quality Assessment

Across all studies, the mean risk of bias score was 18.4 (SD 1.95) out of a maximum score of 28, which is moderate in quality (Table 2). Only one study, an RCT, received a risk of bias score of “good” (Dabas et al., 2019), with the other four studies receiving scores of “fair” (Feher et al., 1989; Howland et al., 2017; Marshall et al., 2019; Mendelson et al., 2018). Most studies scored poorly on external validity (*M* = 1, out of a maximum score of 3, SD = .70) and the internal validity-confounding category (*M* = 3.4, out of a maximum score of 6, SD = 1.14). There was a notable absence across all studies in reporting a power analysis.

**Table 2:** Risk of bias summary

Author (Year)	Reporting (maximum score = 11)	External validity (maximum score = 3)	Internal validity—bias (maximum score = 7)	Internal validity—confounding (maximum score = 6)	Overall score (maximum score = 28)^a^
Dabas et al. (2019)	9	1	5	5	20 (Good)
Feher et al. (1989)	10	1	4	4	19 (Fair)
Howland et al. (2017)	10	2	5	2	19 (Fair)
Marshall et al. (2019)	10	1	5	3	19 (Fair)
Mendelson et al. (2018)	7	0	5	3	15 (Fair)
Mean Scores	9.2	1	4.8	3.4	18.4
Standard Deviation	1.304	0.707	0.447	1.140	1.95

^a^Downs & Black score ranges for quality levels are: excellent (26–28); good (20–25); fair (15–19); and poor (≤ 14) (Downs & Black, 1998)

### Intervention Characteristics

#### Program Descriptions

None of the studies followed a standardized program such as MBSR, and instead, each used researcher-created, custom programs of mindfulness and/or relaxation techniques (Table 3). Most studies combined multiple skills into their intervention programs, with two studies using only one technique (Howland et al., 2017; Mendelson et al., 2018). Breathing exercises, guided imagery, and meditation were most commonly used (Table 4).

**Table 3:** Description of mindfulness and relaxation programs

Study name	Type of program	Delivery method	Program description	Prescribed frequency
*RCTs*				
Dabas et al. (2019)	Custom, developed by researcher and yoga therapist	Pre-recorded (audio)	Recording of 30 min duration, with deep breathing (5 min), Suksham Vyayam (gentle yoga) (8 min), Anulom-Vilom (alternate nostril breathing) (5 min), Brahmari (humming breath) (5 min), PMR (5 min), and deep breathing (2 min). Shown to IG on the day of enrollment by researchers. Then, played on a laptop in a quiet private room of the NICU between 6:00–6:30 p.m. each night for 10 consecutive days (observed by a researcher)	1 × training session and 1×/day for 10 days
Feher et al. (1989)	Custom, developed and recorded by the senior researcher	Pre-recorded (audio tape)	Audio tape of 20-min duration with PMR followed by guided imagery. PMR involved tightening and relaxing various muscles in the body while taking deep rhythmic breaths. Guided imagery included descriptions of pleasant surroundings, milk flowing in the breasts, and the infant’s warm skin against the mother	At least 1×/day prior to milk expression for 7 days
*Non-RCTs*				
Howland et al. (2017)	Custom, created and recorded by a researcher certified in relaxation guided imagery	Pre-recorded (audio CD)	20-min relaxation guided imagery audio recording. Audio recordings had 3 tracks: (1) Developing a relaxed state, (2) working with difficult feelings, and (3) developing a friendlier feeling toward self. Participants were instructed to listen to one track for 2 weeks before they switched to the next track. During the last 2 weeks of study, participants could choose to listen to whichever track they most preferred	1×/day for 8 weeks
Marshall et al. (2019)	Custom, led by a researcher with MBSR training	In-person & pre-recorded (MP3)	Parents received a 1:1 mindfulness-based 60-min training session taught by a researcher and following a set protocol. A glitter globe was used to demonstrate the concept of mindfulness. Then, parents were guided through 3 mindfulness techniques: (1) Intentional relaxing sighs, to focus on being in the moment and experience the breath, (2) pre-recorded mindful meditations (a body scan and breathing meditation), and (3) calming phrases, which parents selected from a provided list. Parents were provided with calming phrases in an envelope at infant bedside and links to free downloadable mindfulness programs as well as access to MP3 mindfulness meditations on an iPad in the NICU	1 training session and then relaxing sighs and calming phrases 2x/day and meditation 2x/week throughout hospital admission
Mendelson et al. (2018)	Custom, created by researchers after interviewing four mindfulness instructors	Pre-recorded (video & MP3)	One 20-min introductory video; then, 1 of 4 mindfulness practice recordings via MP3, each available in 5- and 10-min versions: (1) Following the breath and paying attention without judgment, (2) noticing body sensations, emotions, and state of mind without judgment, (3) sending kindness to another person and to oneself, and (4) identifying emotional pain	One 20-min training session and then participant choice for 2 weeks, with frequent follow-up by research team

*min *minutes, *PMR* progressive muscle relaxation, *MBSR* mindfulness-based stress reduction, *IG* intervention group, *CBT* cognitive behavioral therapy

**Table 4:** Mindfulness and relaxation modalities by study

Mindfulness/relaxation skill	Dabas et al. (2019)***	Feher et al. (1989)*	Howland et al. (2017)	Marshall et al. (2019)	Mendelson et al. (2018)**	Total
Breathing exercises	**✓**	–	–	**✓**	–	2
Progressive muscle relaxation	**✓**	**✓**	–	–	–	2
Guided imagery	–	**✓**	**✓**	–	–	2
Meditation	–	–	–	**✓**	**✓**	2
Gentle stretches	**✓**	–	–	–	–	1
Calming Phrases	–	–	–	**✓**	–	1

*p < .05, **p < .01, ***p < .001

#### Delivery Methods

Delivery methods of interventions ranged from a combination of in-person and pre-recorded materials to fully pre-recorded via video or audio recordings (Table 3). Four studies used pre-recorded intervention delivery exclusively.

#### Intervention Dose, Duration, and Frequency

Intervention dose, duration, and frequency varied widely across studies. Three studies employed intervention sessions of 20-min in length (Feher et al., 1989; Howland et al., 2017; Mendelson et al., 2018) and two ranged from 30- to 60-min (Dabas et al., 2019; Marshall et al., 2019). Participants were asked to use the intervention daily in all studies, and prescribed intervention time periods ranged from 7 days (Feher et al., 1989), to 8 weeks (Howland et al., 2017). One study asked participants to use the intervention throughout their infant’s hospital admission, with an average admission length of 72 days (SD = 42) (Marshall et al., 2019).

Four studies reported data on participants’ compliance with intervention usage recommendations (Feher et al., 1989; Howland et al., 2017; Marshall et al., 2019; Mendelson et al., 2018). Most reported low compliance, with one study reporting that 50% of participants used the intervention at least five times, out of a prescribed minimum of seven times (Feher et al., 1989). Another study reported similar compliance rates, with a mean intervention use of 4.46 times per week (SD = 1.1) instead of the recommended seven times per week (Howland et al., 2017).

### Outcome Measures

There were 19 different measurement scales used across studies, including the Parental Stress Scale: Neonatal Intensive Care Unit (PSS:NICU) and the State–Trait Anxiety Inventory (STAI). The most frequently used scale was the PSS:NICU (Dabas et al., 2019; Marshall et al., 2019; Mendelson et al., 2018). Other measurement tools used across studies included salivary cortisol concentrations (Howland et al., 2017), and expressed breast milk volume (Dabas et al., 2019; Feher et al., 1989).

### Outcome Results

#### Primary Outcomes

In total, three studies reported significant between groups findings on primary outcome measures (Dabas et al., 2019; Feher et al., 1989; Mendelson et al., 2018) (Table 5). Four studies measured stress (Dabas et al., 2019; Howland et al., 2017; Marshall et al., 2019; Mendelson et al., 2018), three measured anxiety (Dabas et al., 2019; Howland et al., 2017; Mendelson et al., 2018), and two measured depression (Howland et al., 2017; Mendelson et al., 2018).

**Table 5:** Summary of results by outcome

Outcome	Study name	Measure	Results (RCT: IG vs. CG; non-RCT: pre- vs. post-intervention)	Intervention summary
Stress (RCTs)				
	Dabas et al. (2019)	PSS:NICU	*M* (SD) = 2.9 (0.5) vs. 3.6 (0.6), *p* = .003, *d* = − 1.27	1×/30 min training and then 1×/30 min multi-modality relaxation-based audio for 10 days
Stress (Non-RCTs)				
	Howland et al. (2017)	PSS	*M* (SD) = 19.55 (5.75) vs. 17.79 (5.80), *p* = > .05,	20 min guided imagery audio recording 1×/day for 8 weeks
	Marshall et al. (2019)	PSS:NICU	MD (SD) = 2.84 (0.34) vs. 2.59 (0.72), *p* = .090, *d* = 0.44	1 × 60 min session, and instructions to use "relaxing sighs" and "calming phrases" 2x/day and meditation (body scan or breathing meditation) 2x/week throughout admission
	Mendelson et al. (2018)	PSS:NICU	MD (SD) = 4.13 (1.19) vs. 3.29 (1.57), *p* = < .05, *d* = 0.60	1 × 20 min session, and instructions to listen to one of 4 meditations available via MP3 1×/day for 2 weeks
Anxiety (RCTs)				
	Dabas et al. (2019)	PASS	*M *(SD) = 19.8 (6.7) vs. 28.18 (11.7), *p* = .003, *d* = − 0.88	1×/30 min training and then 1×/30 min multi-modality relaxation-based audio for 10 days
Anxiety (Non-RCTs)				
	Howland et al. (2017)	STAI	MD (SD) = 42.05 (13.40) vs. 39.42 (12.79), *p* = > .05,	20 min guided imagery audio recording 1×/day for 8 weeks
	Mendelson et al. (2018)	GAD-7	MD (SD) = 8.88 (5.42) vs. 6.04 (4.87), *p* = < 0.05, *d* = − 0.55	1 × 20 min session, and instructions to listen to one of 4 meditations available via MP3 1×/day for 2 weeks
Depression (Non-RCTs)				
	Howland et al. (2017)	CES-D	MD (SD) = 18.45 (11.89) vs. 14.61 (11.79), *p* = > .05,	20 min guided imagery audio recording 1×/day for 8 weeks
	Mendelson et al. (2018)	PHQ-8	*MD* (SD) = 8.54 (4.77) vs. 5.64 (5.62), *p* = < .01, *d* = − 0.55	1 × 20 min session, and instructions to listen to audio MP3 1×/day for 2 weeks
Breastfeeding (RCTs)				
*Milk volume*	Dabas et al. (2019)	mL/express	*M* (SD) = 69.2 mL/express (19.3 mL/express) vs. 54.1 mL/express (22.5 mL/express), *p* = .01, *d* = 0.72	1×/day 30 min multi-modality relaxation-based audio for 11 days
	Feher et al. (1989)	mL/express	Breast milk output *M* (SD) = 90.1 mL/express (60 mL/express) vs. 55.4 mL/express (48.2 mL/express), *p* = < .05, *d* = 0.64	20 min PMR and guided imagery audio recording, before milk expression at least 1×/day
*Breast milk cream content*				
	Feher et al. (1989)	%	*M* (SD) = 7.2% (2.9%) vs. 6.8% (2.4%), *p* = > .05, *d* = 0.15	20 min PMR and guided imagery audio recording, before milk expression at least 1×/day

Effect sizes as reported by study authors or calculated when appropriate data reported

*min* minutes, *RCT* randomized controlled trial, *non-RCT* non-randomized trial, *IG *intervention group, *CG* control group, *PSS:NICU* Parental Stress Scale: Neonatal Intensive Care Unit, *MD* mean difference, *min* minutes, *PSS* Perceived Stress Scale, *PASS* Perinatal Anxiety Screening Scale, *BAI* Beck Anxiety Inventory, *STAI* State-Trait Anxiety Inventory, *GAD-7* General Anxiety Disorder-7, *STAI* State-Trait Anxiety Inventory, *PHQ-8* Patient Health Questionnaire—8, *BDI-II* Beck Depression Inventory, Second Edition, *CES-D* Center for Epidemiologic Studies Depression Scale, *mL/express* milliliters/expressed milk, *PMR* progressive muscle relaxation

Of the four studies that measured stress as a primary outcome, two studies, one RCT (Dabas et al., 2019) and one non-RCT (Mendelson et al., 2018), reported significant reductions between groups, with moderate (*d* = 0.60) (Mendelson et al., 2018) and large effect sizes (*d* = 1.27) (Dabas et al., 2019).

Three studies measured anxiety, and one RCT (Dabas et al., 2019) and one non-RCT (Mendelson et al., 2018) reported significant reductions in anxiety symptoms with effect sizes ranging from moderate (*d* = 0.55) (Mendelson et al., 2018) to large (*d* = 0.88) (Dabas et al., 2019). Additionally, two studies measured depression, and one non-RCT (Mendelson et al., 2018) reported significant reductions in depressive symptoms with a moderate effect size (*d* = 0.55).

#### Secondary Outcomes

Two RCTs assessed breastfeeding measures (Dabas et al., 2019; Feher et al., 1989), and both found significant increases in expressed breast milk quantity after the relaxation-based intervention with moderate effect sizes (*d* = 0.64 and 0.72, respectively). Some studies assessed other outcomes beyond the focus of this review, including trauma symptoms (Mendelson et al., 2018) and mother-infant bonding (Howland et al., 2017).

#### Outcomes by Intervention Delivery Method

Four studies used fully pre-recorded intervention delivery methods (Dabas et al., 2019; Feher et al., 1989; Howland et al., 2017; Mendelson et al., 2018). Of these, three had statistically significant results between groups, with two (Dabas et al., 2019; Mendelson et al., 2018) reporting meaningful results in four outcome measures (stress, anxiety, depression, and expressed breast milk output). Both of these studies used pre-recorded materials to deliver an introductory session to participants; after that, one provided MP3 recordings for participants to use daily (Mendelson et al., 2018) while the other played pre-recorded audio every evening at a scheduled time in the NICU (Dabas et al., 2019). The third study provided a pre-recorded audio tape to participants and reported significant improvements in expressed breast milk output (Feher et al., 1989).

## Discussion

This review systematically assessed whether mindfulness and/or relaxation-based interventions are effective for reducing stress, anxiety, and/or depressive symptoms in parents with infants in the NICU. The evidence presented is mixed, with a diverse range of study designs, intervention programs, and measurement tools used across studies.

The most consistent evidence in this review suggests that mindfulness and/or relaxation-based interventions may be effective at reducing anxiety symptoms in NICU parents, with moderate to large effect sizes. This is similar to previous research, which found MBIs led to moderate to large reductions in anxiety symptoms in perinatal women (Shi & MacBeth, 2017).

The evidence is weaker for the effect on parental stress in the NICU, with one RCT (Dabas et al., 2019) and one non-RCT (Mendelson et al., 2018) out of four studies (Dabas et al., 2019; Howland et al., 2017; Marshall et al., 2019; Mendelson et al., 2018) reporting significant reductions. A variety of factors may have influenced these results, including the use of the PSS:NICU scale in three out of four of these studies (Dabas et al., 2019; Marshall et al., 2019; Mendelson et al., 2018), which measures stress specific to the NICU-environment and does not measure potential effects of the intervention on sources of stress outside of the hospital.

The results of mindfulness and/or relaxation-based interventions on depression are also mixed. Out of two studies that included depression as a primary outcome (Howland et al., 2017; Mendelson et al., 2018), one non-RCT (Mendelson et al., 2018) reported significant reductions in depressive symptoms with moderate effect sizes. Previous studies in other settings have supported the use of MBIs to treat depression, finding MBI programs to be effective at reducing depressive symptoms in postpartum mothers (Pan et al., 2019) and the general adult population (Hofmann et al., 2010). Therefore, further study is merited to assess the effectiveness of mindfulness and/or relaxation-based interventions on parental depressive symptoms in the NICU.

This review shows initial support for the use of relaxation-based interventions to increase expressed breast milk output. The two RCTs that assessed this secondary outcome (Dabas et al., 2019; Feher et al., 1989) reported significant increases in expressed breast milk quantity after intervention use with moderate effect sizes. This finding aligns with previous research that has suggested psychological distress hinders the body’s breast milk “let-down” reflex, and reductions in maternal distress may improve breastfeeding outcomes (Mohd Shukri et al., 2018). This is of importance as premature infants who receive human milk have lower risks of short- and long-term adverse outcomes, including developmental and neurocognitive delays, chronic lung disease, and rehospitalization after NICU discharge (Meier et al., 2010).

Mindfulness and relaxation-based interventions were of interest in this review in part because they are relatively simple to learn, can take as little as 10-min per day to perform, and may not require extensive training to deliver. These are important considerations in the NICU, where parents and staff are often time-constrained and under significant stress (Mendelson et al., 2017). In this review, four studies used a pre-recorded delivery method exclusively, with no intervention dose lasting longer than 30 min (Dabas et al., 2019; Feher et al., 1989; Howland et al., 2017; Mendelson et al., 2018). Notably, three of these studies reported significant results across four outcome categories (stress, anxiety, depression, and expressed breast milk output) (Dabas et al., 2019; Feher et al., 1989; Mendelson et al., 2018). These findings are consistent with previous research showing electronic MBIs had a small but significant effect on reducing depressive and anxiety symptoms and a moderate effect on lowering stress in adult populations (Spijkerman et al., 2016).

### Implications for Clinical Psychology

This review highlights the need to support parents with infants in the NICU, who are significantly stressed and at higher risk of developing anxiety, depression, and acute stress disorders than other parents (Sabnis et al., 2019). The findings of this review suggest mindfulness and relaxation-based interventions show promise in reducing some forms of NICU parent distress, particularly anxiety. It also suggests that relaxation-based interventions may help improve breastfeeding outcomes in NICU mothers.

Importantly, these findings highlight that these types of interventions are feasible to deliver in the NICU setting and acceptable to parents. Brief, pre-recorded interventions, requiring low staff involvement, were found to deliver clinically significant reductions in symptoms. Therefore, this review provides preliminary support for incorporating mindfulness and/or relaxation-based interventions, including pre-recorded versions, into NICU programs to support parents.

### Limitations and Future Research

While promising, this review’s findings should be interpreted with caution in light of a few limitations. Every study in this review used a unique, author-developed intervention program, often incorporating a range of skills, and these factors make them difficult to compare. In the future, the repeated study of manualized interventions is needed to clearly demonstrate that mindfulness and/or relaxation-based interventions are effective with NICU parents. It is also important for researchers to more clearly define the differences between MBIs and relaxation-based interventions to assess the individual effectiveness of each. Additionally, attrition rates in NICU parent studies are typically high, with average rates of 15% or more attrition (Mendelson et al., 2017), and this review found similar results (5% to 29%, *M* = 16%). Reporting of participants’ frequency of use of interventions across studies was also inconsistent, making it difficult to assess dosage effect.

More broadly, most studies in this review were conducted among mothers, limiting the generalizability across the parent population. This is a wider problem across studies on NICU parents, with the majority of research conducted with mothers (Sabnis et al., 2019). However, the limited research on fathers has shown that they experience similar levels of distress as mothers in the NICU (Noergaard et al., 2018; Prouhet et al., 2018), and it is promising that the study that included fathers in this review reported significant reductions in anxiety symptoms and stress levels (Dabas et al., 2019). Future mindfulness and relaxation-based studies should aim to recruit more fathers to evaluate differences in responsiveness and effectiveness.

Similarly, race or ethnicity was only reported in three of these studies (Howland et al., 2017; Marshall et al., 2019; Mendelson et al., 2018), and within those, the majority of participants were Caucasian. Because of significant gaps in data about race and ethnicity in this study group, it is difficult to generalize these findings across diverse cultures. It is also important to note that these studies relied overwhelmingly on self-report screening tools to measure outcomes, and research has found these types of tools can have problematic variations between cultures (Owais et al., 2020).

Study design was also variable in quality. While two studies were RCTs, considered the gold standard of experimental design (Armour et al., 2018), three studies included in this review used a pre-post design with the same group of parents. Using only one group of parents, few conclusions about the effect of the intervention can be drawn as stress levels are expected to reduce with time (Cavaleri et al., 2018). Additionally, the sample size was generally small across these studies, and none reported using a power calculation.

While the literature is growing on the topic of mindfulness and relaxation-based interventions in the NICU, it is still a small body of mostly pilot research. Therefore, it is recommended that future research (1) tests standardized and replicable interventions of this type on parental outcomes (such as stress, anxiety, and depression) in the NICU, (2) evaluate mindfulness and relaxation-based interventions with diverse parent populations, including fathers and those from a range of cultural backgrounds (3) improve study quality with more rigorous study design (e.g., RCTs, the use of consistent and validated measures, and adequately powered sample sizes), and (4) provide standardized outcome measurements during NICU admission as well as after discharge (e.g. three months, six months) to assess long-term effects.

To summarize, the evidence presented in this review is mixed, but shows promise in the effectiveness of mindfulness and/or relaxation-based interventions to reduce some measures of parental distress in the NICU, particularly anxiety, and to improve breastfeeding outcomes for mothers with infants in the NICU. Further studies are needed to explore whether these types of interventions are effective in reducing depressive symptoms and parental stress. Additionally, more rigorous research is needed to determine what delivery methods (e.g., in-person vs. pre-recorded) and specific modalities of mindfulness or relaxation techniques (e.g., PMR, meditation, deep breathing) are most effective to reduce distress for NICU parents.

## Data Availability

Downs & Black Checklist assessments for each study are available as supplementary materials.

## Full Search Strategy

Ovid MEDLINE(R) Epub Ahead of Print, In Process & Other Non-Indexed Citations, Ovid MEDLINE (R) Daily, and Ovid MEDLINE (R) 1946-Present.

1. Mindfulness/
2. mindful*.ti,ab,kw,kf.
3. (mbsr or mbct).ti,ab,kw,kf.
4. Relaxation Therapy/
5. (relax* adj2 (technique* or therap*)).ti,ab,kw,kf.
6. Meditation/
7. meditation.ti,ab,kw,kf.
8. “Acceptance and Commitment Therapy”/
9. (acceptance adj2 commitment therap*).ti,ab,kw,kf.
10. Imagery, Psychotherapy/
11. ((guided or reverie or psychotherap*) adj2 (imag* or therap*)).ti,ab,kw,kf.
12. Autogenic Training/
13. progressive muscle relax*.ti,ab,kw,kf.
14. autogenic training.ti,ab,kw,kf.
15. or/1–14
16. Intensive Care Units, Neonatal/
17. ((neonatal or newborn or neo-natal or preterm or neonate) adj1 (intensive care or ICU?)).ti,ab,kw,kf.
18. NICU.ti,ab,kw,kf.
19. Intensive Care, Neonatal/
20. or/16–19
21. 15 and 20

APA PsycInfo < 1806 to March Week 4 2021 > 

1. Mindfulness/
2. mindful*.ti,ab.
3. (mbsr or mbct).ti,ab.
4. Relaxation Therapy/
5. (relax* adj2 (technique* or therap*)).ti,ab.
6. Meditation/
7. meditation.ti,ab.
8. “Acceptance and Commitment Therapy”/
9. (acceptance adj2 commitment therap*).ti,ab.
10. Imagery, Psychotherapy/
11. ((guided or reverie or psychotherap*) adj2 (imag* or therap*)).ti,ab.
12. Autogenic Training/
13. progressive muscle relax*.ti,ab.
14. autogenic training.ti,ab.
15. or/1–14
16. Intensive Care Units, Neonatal/
17. ((neonatal or newborn or neo-natal or preterm or neonate) adj1 (intensive care or ICU?)).ti,ab.
18. NICU.ti,ab.
19. neonatal intensive care/
20. or/16–19
21. 15 and 20

Embase < 1980 to 2021 March Week 4 2021 > 

1. Mindfulness/
2. mindful*.ti,ab,kw.
3. (mbsr or mbct).ti,ab,kw.
4. Relaxation Therapy/
5. (relax* adj2 (technique* or therap*)).ti,ab,kw.
6. Meditation/
7. meditation.ti,ab,kw.
8. “Acceptance and Commitment Therapy”/
9. (acceptance adj2 commitment therap*).ti,ab,kw.
10. Imagery, Psychotherapy/
11. ((guided or reverie or psychotherap*) adj2 (imag* or therap*)).ti,ab,kw.
12. Autogenic Training/
13. progressive muscle relax*.ti,ab,kw.
14. autogenic training.ti,ab,kw.
15. or/1–14
16. Intensive Care Units, Neonatal/
17. ((neonatal or neonate or newborn or preterm or neo-natal) adj1 (intensive care or ICU?)).ti,ab,kw.
18. NICU.ti,ab,kw.
19. Intensive Care, Neonatal/
20. or/16–19
21. 15 and 20

## SCOPUS

(TITLE-ABS-KEY (mindfulness OR “mbsr” OR “mbct” OR “Relaxation Therapy” OR meditation OR “Acceptance and Commitment Therapy” OR “Autogenic Training” OR “progressive muscle relaxation”) AND TITLE-ABS-KEY (“neonatal intensive care unit” OR “NICU” OR “neonatal intensive care units” OR “newborn intensive care” OR neonate OR preterm)).

### Cochrane Library

- MeSH descriptor: [Intensive Care, Neonatal] explode all trees.
- MeSH descriptor: [Mindfulness] explode all trees.
- MeSH descriptor: [Mind–Body Therapies] explode all trees.
- Mindfulness based stress reduction*
- Mindfulness based*
- mbsr* or mbct*
- MeSH descriptor: [Meditation] explode all trees.
- meditation*
- MeSH descriptor: [Relaxation Therapy] explode all trees.
- (relaxation* near/2 (technique* or therap*)).
- MeSH descriptor: [Intensive Care Units, Neonatal] explode all trees.

### CINAHL Plus

(Mindfulness or mindful* or (mbsr) or (mbct) or (Relaxation Therapy) or ((relax* adj2 (technique* or therap*)) or meditation or (“Acceptance and Commitment Therapy”) or (Imagery, Psychotherapy) or ((guided or reverie or psychotherap*) adj2 (imag* or therap*)) or (Autogenic Training) or (progressive muscle relax*)) AND ((neonatal intensive care unit) or (NICU) or (Intensive Care Units, Neonatal) or ((neonatal or newborn or neo-natal) adj1 (intensive care or ICU?)) or (Intensive Care, Neonatal)).
